# The Spatiotemporal Dynamics of Insect Predator–Prey System Incorporating Refuge Effect

**DOI:** 10.3390/e26030196

**Published:** 2024-02-25

**Authors:** Huayong Zhang, Xiaotong Yuan, Hengchao Zou, Lei Zhao, Zhongyu Wang, Fenglu Guo, Zhao Liu

**Affiliations:** 1 Research Center for Engineering Ecology and Nonlinear Science, North China Electric Power University, Beijing 102206, China; 2 Theoretical Ecology and Engineering Ecology Research Group, School of Life Sciences, Shandong University, Qingdao 250100, China; 3 Beijing Key Laboratory of Biodiversity and Organic Farming, College of Resources and Environmental Sciences, China Agricultural University, Beijing 100193, China

**Keywords:** insect predator–prey system, spatiotemporally discrete, bifurcation, chaos, refuge effect

## Abstract

The insect predator–prey system mediates several feedback mechanisms which regulate species abundance and spatial distribution. However, the spatiotemporal dynamics of such discrete systems with the refuge effect remain elusive. In this study, we analyzed a discrete Holling type II model incorporating the refuge effect using theoretical calculations and numerical simulations, and selected moths with high and low growth rates as two exemplifications. The result indicates that only the flip bifurcation opens the routes to chaos, and the system undergoes four spatiotemporally behavioral patterns (from the frozen random pattern to the defect chaotic diffusion pattern, then the competition intermittency pattern, and finally to the fully developed turbulence pattern). Furthermore, as the refuge effect increases, moths with relatively slower growth rates tend to maintain stability at relatively low densities, whereas moths with relatively faster growth rates can induce chaos and unpredictability on the population. According to the theoretical guidance of this study, the refuge effect can be adjusted to control pest populations effectively, which provides a new theoretical perspective and is a feasible tool for protecting crops.

## 1. Introduction

Insects are rapid adaptive organisms with various magical dynamic behaviors, which can be diametrically different between populations with high and low growth rates [1,2]. Many insects have nonoverlapping generations, shaping discrete dynamics [3,4]. The dynamics of insect populations are not only influenced by various abiotic factors, such as temperature [5,6], altitude [7], and humidity [8], but also largely mediated by predator–prey interactions [9,10]. Refuge effects, a vital influence factor in the insect predator–prey systems [11,12,13], provides a degree of protection for insect populations from predation [11,14,15]. Such spatiotemporally discrete systems are complex, sensitive and prone to chaos [16,17,18,19], whose fluctuations in species abundance or growth rate tend to occur simultaneously across spatial sites [20,21], leading to elusive spatial and temporal dynamics [22,23].

A series of experiments have shown that predator–prey interactions among insects (e.g., moths and spiders) conform to the Holling type II functional response [24,25,26]. In most prior studies, the Holling type II predator–prey models were continuous and analyzed by phase plane and bifurcation diagrams, without integrating ecological phenomenon to analyze dynamic behaviors [25,26,27]. Although some scholars discretized the Holling type II model, the chaotic phase plane diagrams failed to be further distinguished [28,29,30]. These gaps have largely hindered our explorations of the complexity in insect predator–prey systems, thus further studies on the discrete Holling type II models are urgently needed.

In recent years, extensive studies have incorporated the refuge effect into the predator–prey model, confirming the important role of refuge [27,31,32,33]. The continuous Lotka–Volterra predator–prey models show that the refuge effect has a profound effect on predator–prey interaction [34]. Moreover, the predator–prey models with ratio dependence also demonstrate that the refuge effect supports the coexistence of the predator and prey [35]. Notably, the discrete-time predator–prey system, which is more compatible in describing the dynamics of insects than the continuous system considering the generation nonoverlapping of many insects [3,24,25,26], also confirms the stabilizing effect of refuge in predator–prey systems [35,36]. However, the spatiotemporal dynamics of discrete insect predator–prey systems with the refuge effect are still unclear.

In this study, we chose two sets of moth–spider parameters, and then discretized the predator–prey model with a modified Leslie–Gower and Holling type II functional response incorporating the refuge effect [37] to reflect the spatiotemporal dynamics of insect predator–prey systems with relatively lower and higher growth rates. This study is organized as follows: (1) discretize the continuous systems based on coupled image lattice models; (2) apply the center manifold theorem and bifurcation theorems to get the generation conditions of the flip bifurcation and Neimark–Sacker bifurcation; (3) analyze the refuge effect on the spatiotemporal development behavior in moth and spider populations under various types of simulation results, such as bifurcation results, space–time development and spatial amplitude. Our study is intended to reveal the spatiotemporal dynamics of the insect predator–prey system with the refuge effect, which should provide a theoretical basis for future biological control of pests.

## 2. Models and Methods

### 2.1. Spatiotemporally Discrete Model

In this study, we investigate the modified Leslie–Gower and Holling type II functional response predator–prey model, which incorporates refuge effect with protection for a certain percentage of prey [37]:

(1) $$\left\{\begin{array}{l} \frac{\partial H}{\partial t}=H\left(r-aH\right)-\frac{\left(1-m\right)HP}{b+\left(1-m\right)H}\\ \frac{\partial P}{\partial t}=P\left(d-\frac{cP}{b+\left(1-m\right)H}\right) \end{array}\right.$$

In which H and P describe prey and predator population densities at time t; r represents the growth rate of prey; *a* represents the intra-specific competition coefficient of prey; mH represents the role of refuge in the protection of prey, with m representing the proportion of prey protected, $m\epsilon \left[0,\left.1\right]\right.;H/\left(b+H\right)$ represents the Holling type II functional response; d represents the maximum growth rate of predator; $cP/\left(b+H\right)$ represents the modified Leslie–Gower-type numerical response function; c represents the maximum value of the per capita reduction in prey due to the predator; b measures the extent to which the environment provides protection (prey and predator); and r,a,b,c,d are taken as positive constants (see the first table in the Section 2.4).

In reality, refuge comes in all sizes, with smaller refuge only protecting a certain percentage of prey. On this basis, Equation (1) considers the following cases: when m=0, Model (1) represents there being no the refuge here; when m=1, Model (1) represents that the refuge here protects all prey; and when 0<m<1, Model (1) represents that the refuge protects a certain percentage of prey.

In order to observe richer dynamics in the insect–predator system, we add the space term to Model (1), i.e.,

(2) $$\left\{\begin{array}{l} \frac{\partial H}{\partial t}=H\left(r-aH\right)-\frac{\left(1-m\right)HP}{b+\left(1-m\right)H}+D_{1}\nabla^{2}H\\ \frac{\partial P}{\partial t}=P\left(d-\frac{cP}{b+\left(1-m\right)H}\right)+D_{2}\nabla^{2}P \end{array}\right.$$

In which $D_{1}$ and $D_{2}$ describe the diffusion coefficient of the predation system and $\nabla^{2}$ describes the Laplace operator in two dimensions.

Considering that refuge is spatially distributed in patches, we discretize the model (2). Firstly, the space is divided into N×N grid units, in which t is the time interval and h is the space interval. Each grid represents a spatial patch, which does energy exchange with surrounding patches. By applying the spatiotemporally discrete lattice of coupled images, we derive the spatiotemporally discrete predator–prey system, i.e.,

(3) $$\left\{\begin{array}{c} H^{\prime }\left(i,j,t\right)=H\left(i,j,t\right)+\frac{\tau }{\delta^{2}}D_{1}{\nabla_{d}}^{2}H\left(i,j,t\right)\\ P^{\prime }\left(i,j,t\right)=P\left(i,j,t\right)+\frac{\tau }{\delta^{2}}D_{2}{\nabla_{d}}^{2}P\left(i,j,t\right) \end{array}\right.,$$

(4) $$\left\{\begin{array}{c} H\left(i,j,t+1\right)=f\left(H\left(i,j,t\right)\right)=H^{\prime }\left(i,j,t\right)+\tau f_{1}\left(H^{\prime }\left(i,j,t\right),P^{\prime }\left(i,j,t\right)\right)\\ P\left(i,j,t+1\right)=f\left(P\left(i,j,t\right)\right)=P^{\prime }\left(i,j,t\right)+\tau f_{2}\left(H^{\prime }\left(i,j,t\right),P^{\prime }\left(i,j,t\right)\right) \end{array}\right.,$$

(5) $$\left\{\begin{array}{c} {\nabla_{d}}^{2}H\left(i,j,t\right)=H\left(i,j+1,t\right)+H\left(i,j-1,t\right)+H\left(i-1,j,t\right)+H\left(i+1,j,t\right)-4H\left(i,j,t\right)\\ {\nabla_{d}}^{2}P\left(i,j,t\right)=P\left(i,j+1,t\right)+P\left(i,j-1,t\right)+P\left(i-1,j,t\right)+P\left(i+1,j,t\right)-4P\left(i,j,t\right) \end{array}\right.,$$

where $H\left(i,\;j,\;t\right)$ and $P\left(i,\;j,\;t\right)\left(i,j\in \left\{1,2,3,\cdots ,n\right\},t\in Z^{+}\right)$ represent the prey and predator densities at time *t* at the $\left(i,\;j\right)$ lattice point; $H^{\prime }\left(i,\;j,\;t\right)$ and $P^{\prime }\left(i,\;j,\;t\right)$ represent the post-diffusion prey and predator densities; τ and δ describe the spatiotemporally discrete time and space intervals; $\nabla_{d}^{2}$ is the discrete Laplace operators; and $f_{1}$ and $f_{2}$ are functions that are determined by intra- and inter-species interactions between prey and predator, which are as follows:

(6) $$\left\{\begin{array}{l} f_{1}\left(H,P\right)=H\left(r-aH\right)-\frac{\left(1-m\right)HP}{b+\left(1-m\right)H}\\ f_{2}\left(H,P\right)=P\left(d-\frac{cP}{b+\left(1-m\right)H}\right) \end{array}\right.$$

The specific description of a spatiotemporally discrete model incorporating the refuge effect is shown by (2)~(6). In this discrete model, some patches can be randomly selected among N×N patches to indicate refuge of prey, in which we apply Equation (1). This model can reflect some real phenomena and have practical relevance, which lays the groundwork for the parsing analysis for the future steps.

### 2.2. Parsing Analysis Methods

#### 2.2.1. Stability Analysis

Local stability analysis on the fixed points of a spatiotemporally discrete predator–prey system is conducted. According to the natural tools of local stability [38,39], we first remove the spatial terms in Model (2) and add Equation (6) into Equation (4), resulting in the following map expression:

(7) $$\left(\begin{array}{l} H\\ P \end{array}\right)\to \left(\begin{array}{l} H+\tau \left[H\left(r-aH\right)-\frac{\left(1-m\right)HP}{b+\left(1-m\right)H}\right]\\ P+\tau \left[P\left(d-\frac{cP}{b+\left(1-m\right)H}\right)\right] \end{array}\right).$$

According to the definition of the fixed points for the map [40], i.e., calculating

(8) $$\left\{\begin{array}{l} H=H+\tau \left(H\left(r-aH\right)-\frac{\left(1-m\right)HP}{b+\left(1-m\right)H}\right)\\ P=P+\tau \left[P\left(d-\frac{cP}{b+\left(1-m\right)H}\right)\right] \end{array}\right..$$

Therefore, four non-negative fixed points are calculated: $\left(H,P)\to E_{0}(0,0\right)$, $\left(H,P)\to E_{1}(r/a,0\right)$, $\left(H,P)\to E_{2}(0,bd/c\right)$, $\left(H,P)\to E_{3}=(H^{*},P^{*})=((dm-d+cr)/ac,d[b+(1-m)H^{*}]/c)=((dm-d+cr)/ac,\{abcd-d(m-1)[cr+d(m-1)]\}/ac^{2}\right)$.

**Lemma** **1.** *Let* $\lambda_{1}$ *and* $\lambda_{2}$ *be two eigenvalues of a* 2×2 *Jacobian matrix. (a) When* $\left|\lambda_{1}\right|<1$ *and* $\left|\lambda_{2}\right|<1$*, the corresponding fixed point is stable and (b) when* $\left|\lambda_{1}\right|>1$ *or* $\left|\lambda_{2}\right|>1$*, then the corresponding fixed point is unstable.*

**Theorem** **1.** *(a) The fixed point* $E_{0}$ *and* $E_{1}$ *are unstable under any parameter condition; (b) the fixed point* $E_{2}$ *is not stable when* r>(1−m)d/c*; (c) the fixed point* $E_{3}$ *is stable when* q<1 *and* $-\left(1+q\right)<p<1+q$*, where*

(9) $$p=-2+\tau \left\{2aH^{*}-r-d+\frac{2bcP^{*}+\left(2cH^{*}+b\right)\left(1-m\right)P^{*}}{{\left[b+\left(1-m\right)H^{*}\right]}^{2}}\right\},$$

(10) $$\begin{array}{cc} q= & 1+\tau \left\{r+d-2aH^{*}-\frac{2bcP^{*}+\left(2cH^{*}+b\right)\left(1-m\right)P^{*}}{{\left[b+\left(1-m\right)H^{*}\right]}^{2}}\right\}\\ & +\tau^{2}\left(rd-2adH^{*}+\frac{2c\left(2aH^{*}-r\right)P^{*}}{b+\left(1-m\right)H^{*}}-\frac{bd\left(1-m\right)P^{*}}{{\left(b+\left(1-m\right)H^{*}\right)}^{2}}\right)\\ & +\tau^{2}\left(\frac{c\left(1-m\right)\left(2b+\left(1-m\right)H^{*}\right){\left(P^{*}\right)}^{2}}{{\left(b+\left(1-m\right)H^{*}\right)}^{3}}\right). \end{array}$$

**Proof.**  The Jacobian matrix of Map (7) can be analyzed for the stability of fixed points [38,39] and its expression at any point is

(11) $$J\left(E_{i}\right)=\left(\begin{array}{cc} 1+\tau \left\{r-2aH-\frac{b\left(1-m\right)P}{{\left[b+\left(1-m\right)H\right]}^{2}}\right\} & \frac{\tau \left(m-1\right)H}{b+\left(1-m\right)H}\\ \frac{\tau c\left(1-m\right)P^{2}}{{\left[b+\left(1-m\right)H\right]}^{2}} & 1+\tau \left[d-\frac{2cP}{b+\left(1-m\right)H}\right] \end{array}\right),\;i=0,1,2,3.$$

The eigenvalues of the Jacobian matrices $J\left(E_{0}\right)$ and $J\left(E_{1}\right)$ of the fixed point $E_{0}$ and $E_{1}$ are $\lambda_{1}=1+\tau r$ and $\lambda_{2}=1+\tau r$. Since $\lambda_{2\;}>1$, the fixed points ${\;E}_{0}$ and $E_{1}$ are unstable according to Lemma 1.

In addition, the eigenvalues of the Jacobian matrix $J\left(E_{2}\right)$ of the fixed point $E_{2}$ is $\lambda_{1}=$$1+\tau [r-\left(1-m\right)d/c]$ and $\lambda_{2}=1-\tau d$, so when r>(1−m)d/c, the fixed point $E_{2}$ is unstable.

Similarly, the eigenvalues of the Jacobian matrix $J\left(E_{3}\right)$ of the fixed point $E_{3}$ are

(12) $$\lambda_{1,2}=\frac{-p\pm \sqrt{p^{2}-4q}}{2}.$$

When q<1 and $-\left(1+q\right)<p<1+q$, there is $\left|\lambda_{1}\right|<1$ and $\left|\lambda_{2}\right|<1$, by Lemma 1 the fixed point $E_{3}$ is stable. □

**Figure 1:**
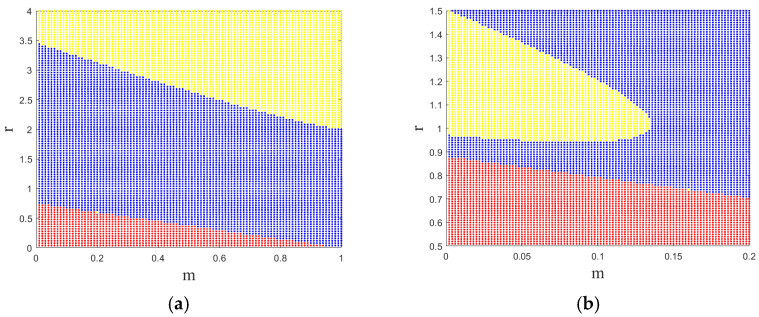
(**a**) Parameter space diagram (m,r) of $E_{1},E_{2},and\;E_{3}$ under a=0.8, b=0.9, c=0.44, d=0.33, (**b**) parameter space diagram (m,r) of $E_{1},E_{2}\;and\;E_{3}$ under a=0.6, b=0.16, c=0.42, d=0.37 (Red represents stable region of $E_{2}$, blue represents stable region of $E_{3}$, and yellow represents unstable region of $E_{1},E_{2},and\;E_{3}$).

Figure 1 illustrates the parameter space diagram of the fixed points $E_{1},E_{2},\;and\;E_{3}$ under two sets of parameters. Moreover, from Theorem 1, when $E_{2}$ is stable, the discrete predator–prey system will converge to the state where the predator is extinct and prey exists only. The extinction of the predator in this state suggests the predator–prey interactions disappear, which leads to no pattern formation. Therefore, we merely focus on the pattern formation at the stable point $E_{3}$.

According to the center manifold theorem [41,42] and the bifurcation theorems, we conduct a bifurcation analysis of spatiotemporally discrete predator–prey systems around the stable point $E_{3}$ using the refuge effect parameter *m* as the bifurcation parameter. Then, we determine the generate conditions of flip bifurcation and Neimark–Sacker bifurcation, respectively.

#### 2.2.2. Flip Bifurcation Analysis

According to the flip bifurcation theorem [41,43], the flip bifurcation occurs when one of the two eigenvalues of $J\left(E_{3}\right)$ satisfies λ=−1. If give q=p−1, in other words,

(13) $$A_{1}{\left(m-1\right)}^{3}+A_{2}{\left(m-1\right)}^{2}+A_{3}\left(m-1\right)+A_{4}=0$$

in which

(14) $$\begin{array}{cc} A_{1}= & \left(2\tau^{2}ad+4\tau a\right){\left(H^{*}\right)}^{4}-\left(2\tau r+2\tau d+\tau^{2}dr+4\right){\left(H^{*}\right)}^{3},\\ A_{2}= & \left(12\tau ab+6\tau^{2}abd-4\tau^{2}a\right){\left(H^{*}\right)}^{3}-\left(12b+6\tau br+6\tau bd+3\tau^{2}bdr+2cP^{*}-2\tau^{2}rP^{*}\right){\left(H^{*}\right)}^{2}\\ & -\left[\tau^{2}bdP^{*}+bP^{*}+\tau^{2}c{\left(P^{*}\right)}^{2}\right]H^{*},\\ A_{3}= & \left(12\tau ab^{2}+6\tau^{2}ab^{2}d-8\tau^{2}ab\right){\left(H^{*}\right)}^{2}-\left(12b^{2}+6\tau b^{2}r+6\tau b^{2}d+3\tau^{2}b^{2}dr-4\tau^{2}brP^{*}-2bcP^{*}\right)H^{*}\\ & +\tau b^{3}dP^{*}+b^{2}P^{*}-2\tau^{2}bc{\left(P^{*}\right)}^{2},\\ A_{4}= & \left(4\tau ab^{3}+2\tau^{2}ab^{3}d-4\tau^{2}ab^{2}\right)H^{*}+2\tau^{2}b^{2}rP^{*}+2\tau b^{2}cP^{*}-4b^{3}-2\tau b^{3}r-2\tau b^{3}d-\tau^{2}b^{3}dr. \end{array}$$

According to the Cardano formula, the above one-dimensional cubic Equation (13) can be equated to

(15) $${\left(m-1\right)}^{3}+k_{1}\left(m-1\right)+k_{2}=0.$$

Then can be obtained as

(16) $$m^{*}=\sqrt[{3}]{-\frac{k_{2}}{2}+\sqrt{\frac{{k_{1}}^{3}}{27}+\frac{{k_{2}}^{2}}{4}}}+\sqrt[{3}]{-\frac{k_{2}}{2}-\sqrt{\frac{{k_{1}}^{3}}{27}+\frac{{k_{2}}^{2}}{4}}}+1$$

In which

(17) $$\begin{array}{l} k_{1}=\frac{3A_{3}-A_{2}}{3A_{1}},\\ k_{2}=\frac{{A_{2}}^{3}-3A_{2}A_{3}+9{A_{1}}^{2}A_{4}}{9{A_{1}}^{3}}. \end{array}$$

With the above conditions (16) satisfied, which leads to the two eigenvalues of ${J(E}_{3})$ become $\lambda_{1}=-1$ and $\lambda_{2}=1-p(m^{*})$, and the generation of the flip bifurcation requires the satisfaction $\left|\lambda_{2}\right|\neq 1$, i.e.,

(18) $$\tau \left(2aH^{*}-r-d+\frac{2bcP^{*}+\left(2cH^{*}+b\right)\left(1-m^{*}\right)P^{*}}{{\left(b+\left(1-m^{*}\right)H^{*}\right)}^{2}}\right)\neq 2,4.$$

When Equation (16) is satisfied, let

(19) $$w=H-H*,\;z=P-P*,\;\tilde{m}=m-m^{*},$$

By translating the fixed point of map (7) to the origin and performing a Taylor expansion near the fixed point $E_{3}$, we can transform map (7) into the following expression:

(20) $$\left(\begin{array}{l} w\\ z\\ \tilde{m} \end{array}\right)\to \left(\begin{array}{l} a_{11}w+a_{12}z+a_{13}\tilde{m}+\frac{a_{14}}{2}w^{2}+a_{15}wz+a_{16}w\tilde{m}+a_{17}z\tilde{m}+\frac{a_{18}}{2}{\tilde{m}}^{2}+\frac{a_{19}}{6}w^{3}+\frac{a_{110}}{2}w^{2}z\\ +\frac{a_{111}}{2}w^{2}\tilde{m}+a_{112}wz\tilde{m}+\frac{a_{113}}{2}w{\tilde{m}}^{2}+\frac{a_{114}}{2}z{\tilde{m}}^{2}+\frac{a_{115}}{6}{\tilde{m}}^{3}+O\left({\left(\left|\left.w\right|\right.+\left|\left.z\right|\right.+\left|\left.\tilde{m}\right|\right.\right)}^{4}\right)\\ a_{21}w+a_{22}z+a_{23}\tilde{m}+\frac{a_{24}}{2}w^{2}+a_{25}wz+a_{26}w\tilde{m}+a_{27}z\tilde{m}+\frac{a_{28}}{2}z^{2}+\frac{a_{29}}{2}{\tilde{m}}^{2}+\frac{a_{210}}{6}w^{3}\\ +\frac{a_{211}}{2}w^{2}z+\frac{a_{212}}{2}w^{2}\tilde{m}+\frac{a_{213}}{2}wz^{2}+a_{214}wz\tilde{m}+\frac{a_{215}}{2}w{\tilde{m}}^{2}+\frac{a_{216}}{2}z{\tilde{m}}^{2}+\frac{a_{217}}{2}z^{2}\tilde{m}\\ +\frac{a_{118}}{6}{\tilde{m}}^{3}+O\left({\left(\left|\left.w\right|\right.+\left|\left.z\right|\right.+\left|\left.\tilde{m}\right|\right.\right)}^{4}\right)\\ \tilde{m} \end{array}\right)$$

In which $O\left({\left(\left|w\right|+\left|z\right|+\left|\tilde{m}\right|\right)}^{4}\right)$ is a polynomial function of at least fourth order for the dependent variable and

$$a_{11}=1+\tau \left\{r-2aH^{*}-\frac{b\left(1-m\right)P^{*}}{{\left[b+\left(1-m\right)H^{*}\right]}^{2}}\right\},\;a_{12}=\frac{\tau \left(m-1\right)H^{*}}{b+\left(1-m\right)H^{*}},\;a_{13}=\frac{\tau bH^{*}P^{*}}{{\left[b+\left(1-m\right)H^{*}\right]}^{2}},\;a_{14}=-2\tau a+\frac{2\tau b{\left(1-m\right)}^{2}P^{*}}{{\left[b+\left(1-m\right)H^{*}\right]}^{3}},\;a_{15}=-\frac{\tau b\left(1-m\right)}{{\left[b+\left(1-m\right)H^{*}\right]}^{2}},\;a_{16}=\frac{\tau bP^{*}\left[b-\left(1-m\right)H^{*}\right]}{{\left[b+\left(1-m\right)H^{*}\right]}^{3}},\;a_{17}=\frac{\tau bH^{*}}{{\left(b+\left(1-m\right)H^{*}\right)}^{2}},\;a_{18}=\frac{2\tau b{\left(H^{*}\right)}^{2}P^{*}}{{\left[b+\left(1-m\right)H^{*}\right]}^{3}},\;a_{19}=-\frac{6\tau b{\left(1-m\right)}^{3}P^{*}}{{\left[b+\left(1-m\right)H^{*}\right]}^{4}},\;a_{110}=\frac{2\tau b{\left(1-m\right)}^{2}}{{\left[b+\left(1-m\right)H^{*}\right]}^{3}},\;a_{111}=\frac{2\tau b\left(1-m\right)P^{*}\left[\left(1-m\right)H^{*}-2b\right]}{{\left[b+\left(1-m\right)H^{*}\right]}^{4}},\;a_{112}=\frac{\tau b\left[b-\left(1-m\right)H^{*}\right]}{{\left[b+\left(1-m\right)H^{*}\right]}^{3}},\;a_{113}=\frac{2\tau bP^{*}H^{*}\left[2b-\left(1-m\right)H^{*}\right]}{{\left[b+\left(1-m\right)H^{*}\right]}^{3}},\;a_{114}=\frac{2\tau b{\left(H^{*}\right)}^{2}}{{\left[b+\left(1-m\right)H^{*}\right]}^{3}},\;a_{115}=\frac{6\tau b{\left(H^{*}\right)}^{3}P^{*}}{{\left[b+\left(1-m\right)H^{*}\right]}^{4}},\;a_{21}=\frac{\tau c\left(1-m\right){\left(P^{*}\right)}^{2}}{{\left[b+\left(1-m\right)H^{*}\right]}^{2}},\;a_{22}=1+\tau \left[d-\frac{2cP^{*}}{b+\left(1-m\right)H^{*}}\right],\;a_{23}=-\frac{\tau cH^{*}{\left(P^{*}\right)}^{2}}{{\left[b+\left(1-m\right)H^{*}\right]}^{2}},\;a_{24}=-\frac{4\tau c{\left(1-m\right)}^{2}{\left(P^{*}\right)}^{2}}{{\left[b+\left(1-m\right)H^{*}\right]}^{2}},\;a_{25}=\frac{2\tau c\left(1-m\right)P^{*}}{{\left[b+\left(1-m\right)H^{*}\right]}^{2}},\;a_{26}=\frac{\tau c{\left(P^{*}\right)}^{2}\left[\left(1-m\right)H^{*}-b\right]}{{\left[b+\left(1-m\right)H^{*}\right]}^{3}},\;a_{27}=-\frac{2\tau cH^{*}P^{*}}{{\left[b+\left(1-m\right)H^{*}\right]}^{2}},\;a_{28}=-\frac{2\tau c}{b+\left(1-m\right)H^{*}},\;a_{29}=-\frac{2\tau c{\left(H^{*}\right)}^{2}{\left(P^{*}\right)}^{2}}{{\left[b+\left(1-m\right)H^{*}\right]}^{3}},\;a_{210}=\frac{8\tau c{\left(1-m\right)}^{3}{\left(P^{*}\right)}^{2}}{{\left[b+\left(1-m\right)H^{*}\right]}^{2}},\;a_{211}=-\frac{8\tau c{\left(1-m\right)}^{2}P^{*}}{{\left[b+\left(1-m\right)H^{*}\right]}^{2}},\;a_{212}=\frac{8\tau bc\left(1-m\right){\left(P^{*}\right)}^{2}}{{\left[b+\left(1-m\right)H^{*}\right]}^{3}},\;a_{213}=\frac{2\tau c\left(1-m\right)}{{\left[b+\left(1-m\right)H^{*}\right]}^{2}},\;a_{214}=\frac{2\tau cP^{*}\left[\left(1-m\right)H^{*}-b\right]}{{\left[b+\left(1-m\right)H^{*}\right]}^{3}},\;a_{215}=\frac{2\tau c{\left(P^{*}\right)}^{2}H^{*}\left[\left(1-m\right)H^{*}-2b\right]}{{\left[b+\left(1-m\right)H^{*}\right]}^{4}},\;a_{216}=-\frac{4\tau c{\left(H^{*}\right)}^{2}P^{*}}{{\left[b+\left(1-m\right)H^{*}\right]}^{3}},\;a_{217}=-\frac{2\tau cH^{*}}{{\left[b+\left(1-m\right)H^{*}\right]}^{2}},\;a_{218}=-\frac{6\tau c{\left(H^{*}\right)}^{3}{\left(P^{*}\right)}^{2}}{{\left[b+\left(1-m\right)H^{*}\right]}^{4}}.$$

Then, according to Map (20), we get a reversible transformation as follows:

(21) $$\left(\begin{array}{l} \tilde{w}\\ \tilde{z}\\ \tilde{m} \end{array}\right)\to \left(\begin{array}{ccc} -1 & 0 & 0\\ 0 & \lambda_{2} & 0\\ 0 & 0 & 1 \end{array}\right)\left(\begin{array}{l} \tilde{w}\\ \tilde{z}\\ \tilde{m} \end{array}\right)+\frac{1}{a_{12}\left(1+\lambda_{2}\right)}\left(\begin{array}{l} F_{1}\left(\tilde{w},\tilde{z},\tilde{m}\right)\\ F_{2}\left(\tilde{w},\tilde{z},\tilde{m}\right)\\ 0 \end{array}\right)$$

In which

(22) $$\begin{array}{cc} F_{1}\left(\tilde{w},\tilde{z},\tilde{m}\right) & =\frac{a_{12}^{2}}{2}\left[a_{14}\left(\lambda_{2}-a_{11}\right)-a_{12}a_{24}\right]{\left(\tilde{w}+\tilde{z}\right)}^{2}-a_{12}\left[a_{15}\left(\lambda_{2}-a_{11}\right)+a_{12}a_{25}\right]\\ & \left(\tilde{w}+\tilde{z}\right)\left[\left(1+a_{11}\right)\tilde{w}-\left(\lambda_{2}-a_{11}\right)\tilde{z}\right]+a_{12}\left[a_{16}\left(\lambda_{2}-a_{11}\right)-a_{12}a_{26}\right]\\ & \left(\tilde{w}+\tilde{z}\right)\tilde{m}-\left[a_{17}\left(\lambda_{2}-a_{11}\right)+a_{12}a_{27}\right]\left[\left(1+a_{11}\right)\tilde{w}-\left(\lambda_{2}-a_{11}\right)\tilde{z}\right]\tilde{m}\\ & -\frac{a_{12}a_{28}}{2}{\left[\left(1+a_{11}\right)\tilde{w}-\left(\lambda_{2}-a_{11}\right)\tilde{z}\right]}^{2}+\frac{1}{2}\left[a_{18}\left(\lambda_{2}-a_{11}\right)-a_{12}a_{29}\right]{\tilde{m}}^{2}\\ & +\frac{a_{12}^{3}}{6}\left[a_{19}\left(\lambda_{2}-a_{11}\right)-a_{12}a_{210}\right]{\left(\tilde{w}+\tilde{z}\right)}^{3}-\frac{a_{12}^{2}}{2}\left[a_{110}\left(\lambda_{2}-a_{11}\right)-a_{12}a_{211}\right]\\ & {\left(\tilde{w}+\tilde{z}\right)}^{2}\left[\left(1+a_{11}\right)\tilde{w}-\left(\lambda_{2}-a_{11}\right)\tilde{z}\right]+\frac{a_{12}^{2}}{2}\left[a_{111}\left(\lambda_{2}-a_{11}\right)-a_{12}a_{212}\right]\\ & {\left(\tilde{w}+\tilde{z}\right)}^{2}\tilde{m}-\frac{a_{12}^{2}a_{213}}{2}\left(\tilde{w}+\tilde{z}\right){\left[\left(1+a_{11}\right)\tilde{w}-\left(\lambda_{2}-a_{11}\right)\tilde{z}\right]}^{2}-a_{12}\\ & \left[a_{112}\left(\lambda_{2}-a_{11}\right)+a_{12}a_{214}\right]\left(\tilde{w}+\tilde{z}\right)\left[\left(1+a_{11}\right)\tilde{w}-\left(\lambda_{2}-a_{11}\right)\tilde{z}\right]\tilde{m}\\ & +\frac{a_{12}}{2}\left[a_{113}\left(\lambda_{2}-a_{11}\right)-a_{12}a_{215}\right]\left(\tilde{w}+\tilde{z}\right){\tilde{m}}^{2}-\frac{1}{2}\left[a_{114}\left(\lambda_{2}-a_{11}\right)-a_{12}a_{216}\right]\\ & \left[\left(1+a_{11}\right)\tilde{w}-\left(\lambda_{2}-a_{11}\right)\tilde{z}\right]{\tilde{m}}^{2}+\frac{a_{12}a_{217}}{2}{\left[\left(1+a_{11}\right)\tilde{w}-\left(\lambda_{2}-a_{11}\right)\tilde{z}\right]}^{2}\tilde{m}\\ & +\frac{1}{6}\left[a_{115}\left(\lambda_{2}-a_{11}\right)+a_{12}a_{218}\right]{\tilde{m}}^{3}+O\left({\left(\left|\tilde{w}\right|+\left|\tilde{z}\right|+\left|\tilde{m}\right|\right)}^{4}\right), \end{array}$$

(23) $$\begin{array}{cc} F_{2}\left(\tilde{w},\tilde{z},\tilde{m}\right) & =\frac{a_{12}^{2}}{2}\left[a_{14}\left(1+a_{11}\right)+a_{12}a_{24}\right]{\left(\tilde{w}+\tilde{z}\right)}^{2}-a_{12}\left[a_{15}\left(1+a_{11}\right)-a_{12}a_{25}\right]\\ & \left(\tilde{w}+\tilde{z}\right)\left[\left(1+a_{11}\right)\tilde{w}-\left(\lambda_{2}-a_{11}\right)\tilde{z}\right]+a_{12}\left[a_{16}\left(1+a_{11}\right)+a_{12}a_{26}\right]\\ & \left(\tilde{w}+\tilde{z}\right)\tilde{m}-\left[a_{17}\left(1+a_{11}\right)-a_{12}a_{27}\right]\left[\left(1+a_{11}\right)\tilde{w}-\left(\lambda_{2}-a_{11}\right)\tilde{z}\right]\tilde{m}\\ & -\frac{a_{12}a_{28}}{2}{\left[\left(1+a_{11}\right)\tilde{w}-\left(\lambda_{2}-a_{11}\right)\tilde{z}\right]}^{2}+\frac{1}{2}\left[a_{18}\left(1+a_{11}\right)+a_{12}a_{29}\right]{\tilde{m}}^{2}\\ & +\frac{a_{12}^{3}}{6}\left[a_{19}\left(1+a_{11}\right)+a_{12}a_{210}\right]{\left(\tilde{w}+\tilde{z}\right)}^{3}-\frac{a_{12}^{2}}{2}\left[a_{110}\left(1+a_{11}\right)+a_{12}a_{211}\right]\\ & {\left(\tilde{w}+\tilde{z}\right)}^{2}\left[\left(1+a_{11}\right)\tilde{w}-\left(\lambda_{2}-a_{11}\right)\tilde{z}\right]+\frac{a_{12}^{2}}{2}\left[a_{111}\left(1+a_{11}\right)+a_{12}a_{212}\right]\\ & {\left(\tilde{w}+\tilde{z}\right)}^{2}\tilde{m}-\frac{a_{12}^{2}a_{213}}{2}\left(\tilde{w}+\tilde{z}\right){\left[\left(1+a_{11}\right)\tilde{w}-\left(\lambda_{2}-a_{11}\right)\tilde{z}\right]}^{2}-a_{12}\\ & \left[a_{112}\left(1+a_{11}\right)-a_{12}a_{214}\right]\left(\tilde{w}+\tilde{z}\right)\left[\left(1+a_{11}\right)\tilde{w}-\left(\lambda_{2}-a_{11}\right)\tilde{z}\right]\tilde{m}\\ & +\frac{a_{12}}{2}\left[a_{113}\left(1+a_{11}\right)+a_{12}a_{215}\right]\left(\tilde{w}+\tilde{z}\right){\tilde{m}}^{2}-\frac{1}{2}\left[a_{114}\left(1+a_{11}\right)+a_{12}a_{216}\right]\\ & \left[\left(1+a_{11}\right)\tilde{w}-\left(\lambda_{2}-a_{11}\right)\tilde{z}\right]{\tilde{m}}^{2}+\frac{a_{12}a_{217}}{2}{\left[\left(1+a_{11}\right)\tilde{w}-\left(\lambda_{2}-a_{11}\right)\tilde{z}\right]}^{2}\tilde{m}\\ & +\frac{1}{6}\left[a_{115}\left(1+a_{11}\right)-a_{12}a_{218}\right]{\tilde{m}}^{3}+O\left({\left(\left|\tilde{w}\right|+\left|\tilde{z}\right|+\left|\tilde{m}\right|\right)}^{4}\right). \end{array}$$

On the basis of the center manifold theorem [41,42], the center prevalence $W^{C}\left(0,\;0,\;0\right)$ of Map (21) exists at the fixed point $\left(0,\;0,\;0\right)$, which can be approximated as

(24) $$W^{C}\left(0,0,0\right)=\left\{\left(\tilde{w},\tilde{z},\tilde{m}\right)\in R^{3}\left|\tilde{z}=h*\left(\tilde{w},\tilde{m}\right)=e_{0}\tilde{m}+e_{1}\tilde{w}+e_{2}{\tilde{w}}^{2}+e_{3}\tilde{w}\tilde{m}+e_{4}{\tilde{m}}^{2}+O\left({\left(\left|\tilde{w}\right|+\left|\tilde{m}\right|\right)}^{3}\right)\right.\right\}$$

In which

(25) $$\begin{array}{l} e_{0}=0,\\ e_{1}=0,\\ e_{2}=\frac{a_{12}\left(1+a_{11}\right)\left(a_{14}+2a_{25}\right)+{\left(1+a_{11}\right)}^{2}\left(2a_{25}-a_{28}\right)+{a_{12}}^{2}a_{24}}{2\left(1-\lambda_{2}^{2}\right)},\\ e_{3}=\frac{\left[a_{12}a_{25}-\left(1+a_{11}\right)\left(a_{15}+a_{28}\right)\right]\left(\lambda_{2}-a_{11}\right)-a_{12}\left(1+a_{11}\right)\left(a_{14}+a_{25}\right)+a_{15}{\left(1+a_{11}\right)}^{2}-a_{12}^{2}a_{24}}{a_{12}{\left(1+\lambda_{2}\right)}^{2}},\\ e_{4}=\frac{a_{18}\left(1+a_{11}\right)+a_{12}a_{29}}{2\left(1-\lambda_{2}^{2}\right)}. \end{array}$$

Correspondingly, the dynamics of Map (19) restricted to the center manifold $W^{C}\left(0,\;0,\;0\right)$ are obtained, namely:

(26) $$F:\tilde{w}\to -\tilde{w}+\mu_{1}{\tilde{w}}^{2}+\mu_{2}\tilde{w}\tilde{m}+\mu_{3}{\tilde{m}}^{2}+\mu_{4}{\tilde{w}}^{2}\tilde{m}+\mu_{5}\tilde{w}{\tilde{m}}^{2}+\mu_{6}{\tilde{w}}^{3}+O\left({\left(\left|\tilde{w}\right|+\left|\tilde{m}\right|\right)}^{4}\right)$$

where

$$\mu_{1}=\frac{1}{2\left(1+\lambda_{2}\right)}\left\{a_{12}\left[a_{14}\left(\lambda_{2}-a_{11}\right)-a_{12}a_{24}\right]-2\left[a_{15}\left(\lambda_{2}-a_{11}\right)+a_{12}a_{25}\right]\left(1+a_{11}\right)-a_{28}{\left(1+a_{11}\right)}^{2}\right\},\;\mu_{2}=\frac{a_{16}\left(\lambda_{2}-a_{11}\right)-a_{12}a_{26}}{1+\lambda_{2}}-\frac{\left[a_{17}\left(\lambda_{2}-a_{11}\right)+a_{12}a_{27}\right]\left(1+a_{11}\right)}{a_{12}\left(1+\lambda_{2}\right)},\;\mu_{3}=\frac{a_{18}\left(\lambda_{2}-a_{11}\right)-a_{12}a_{29}}{2a_{12}\left(1+\lambda_{2}\right)},\;\begin{array}{l} \mu_{4}=\frac{1}{1+\lambda_{2}}\left\{\left[a_{14}\left(\lambda_{2}-a_{11}\right)-a_{12}a_{24}\right]e_{3}-\left[a_{15}\left(\lambda_{2}-a_{11}\right)+a_{12}a_{25}\right]\left[-e_{3}\left(\lambda_{2}-a_{11}\right)+\left(1+a_{11}\right)e_{3}\right]\right.\\ +\left.\left[a_{16}\left(\lambda_{2}-a_{11}\right)-a_{12}a_{26}\right]e_{2}-a_{28}\left(1+a_{11}\right)\left(\lambda_{2}-a_{11}\right)e_{3}\right\}+\frac{\left[a_{17}\left(\lambda_{2}-a_{11}\right)+a_{12}a_{27}\right]\left(\lambda_{2}-a_{11}\right)e_{2}}{a_{12}\left(1+\lambda_{2}\right)}, \end{array}\;\begin{array}{cc} \mu_{5}= & \frac{1}{1+\lambda_{2}}\left\{a_{12}\left[a_{14}\left(\lambda_{2}-a_{11}\right)-a_{12}a_{24}\right]e_{4}-\left[a_{15}\left(\lambda_{2}-a_{11}\right)+a_{12}a_{25}\right]\right.\left[\left(1+a_{11}\right)e_{3}-\left(\lambda_{2}-a_{11}\right)e_{3}\right]\\ & \left.\left[a_{16}\left(\lambda_{2}-a_{11}\right)-a_{12}a_{26}\right]e_{3}-a_{28}\left(1+a_{11}\right)\left(\lambda_{2}-a_{11}\right)e_{4}\right\}+\frac{1}{a_{12}\left(1+\lambda_{2}\right)}\left[a_{17}\left(\lambda_{2}-a_{11}\right)+a_{12}a_{27}\right]\left(\lambda_{2}-a_{11}\right)e_{3}, \end{array}\;\begin{array}{cc} \mu_{6}= & \frac{1}{a_{12}\left(1+\lambda_{2}\right)}\left\{a_{12}\left[a_{16}\left(\lambda_{2}-a_{11}\right)-a_{12}a_{26}\right]e_{4}+\left[a_{17}\left(\lambda_{2}-a_{11}\right)+a_{12}a_{27}\right]\left(\lambda_{2}-a_{11}\right)e_{2}\right.\\ & \left.+\frac{1}{6}\left[a_{115}\left(\lambda_{2}-a_{11}\right)+a_{12}a_{218}\right]\right\}. \end{array}$$

In addition, the occurrence of a flip bifurcation for Map (20) requires the following two determinants to be nonzero:

(27) $$\eta_{1}=\frac{\partial^{2}F}{\partial \tilde{w}\partial \tilde{m}}+\frac{1}{2}\frac{\partial F}{\partial \tilde{m}}\frac{\partial^{2}F}{\partial {\tilde{w}}^{2}},\eta_{2}=\frac{1}{6}\frac{\partial^{3}F}{\partial {\tilde{w}}^{3}}+{\left(\frac{1}{2}\frac{\partial^{2}F}{\partial {\tilde{w}}^{2}}\right)}^{2}.$$

The calculation gives that $\eta_{1}=\mu_{2}$, $\eta_{2}=\mu_{5}+\mu_{1}^{2}$.

**Theorem** **2.** *In the discrete predator–prey system (4), a flip bifurcation at the fixed point* $\left(H^{*},\;P^{*}\right)$ *occurs if Equations (16) and (18),* $\mu_{2}\neq 0$ *and* $\mu_{5}\neq \mu_{1}^{2}$ *are satisfied. When* $\eta_{2}>0$ *, the period-2 points bifurcating from the* $\left(H^{*},\;P^{*}\right)$ *are stable; when* $\eta_{2}<0$ *, the bifurcating period-2 points are unstable.*

**Figure 2:**
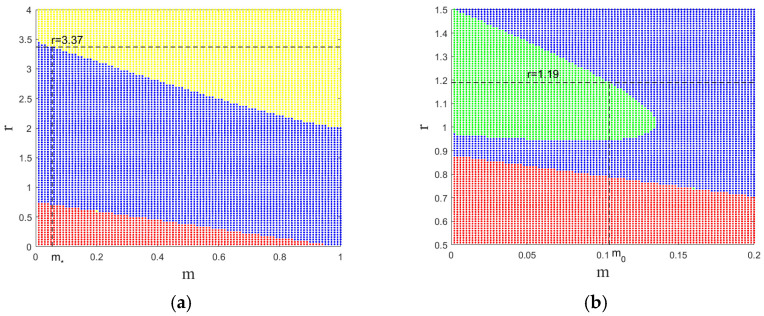
(**a**) Parameter space diagram (m,r) of $E_{1},E_{2},and\;E_{3}$ under a=0.8,b=0.9,c=0.44,d=0.33, (**b**) parameter space diagram (m,r) of $E_{1},E_{2},and\;E_{3}$ under a=0.6, b=0.16, c=0.42, d=0.37 (Around $E_{1},$ the system in all regions has always been unstable and cannot produce bifurcations; around $E_{2}$, the system from the blue region into the red region gradually converges to the state where the predator is extinct; and around $E_{3}$, the system from the blue region into the yellow region is undergoing the flip bifurcation, and from the blue region into the green region is undergoing Neimark–Sacker bifurcation).

The detailed calculation process of Neimark–Sacker bifurcation is shown below. The parameter values are r=3.37,a=0.8,b=0.9,d=0.33,and c=0.44 after calculating the critical value of the occurrence of the flip bifurcation $m^{*}=0.054124$. At this time, the discriminant values are $\eta_{1}=-0.096$ and $\eta_{2}=0.4971$. On the basis of Theorem 2, we can observe that the period-2 orbitals of the system stabilize from the bifurcation of $\left(H^{*},\;P^{*}\right)$. Moreover, from Figure 2a, it can be seen that for the above given parameters, the system is undergoing the flip bifurcation at $m^{*}$.

#### 2.2.3. Neimark–Sacker Bifurcation Analysis

According to the Neimark–Sacker bifurcation theorem [44], Neimark–Sacker bifurcation-generating conditions around the spatiotemporally discrete predator–prey first requires that the eigenvalues in Equation (10) are a pair of conjugate complexes: Modulo 1, i.e., $p^{2}-4q<0$ and q=1, that is to say,

(28) $$\begin{array}{l} {\left(r+d-2aH^{*}-\frac{2bcP^{*}+\left(2cH^{*}+b\right)\left(1-m\right)P^{*}}{{\left(b+\left(1-m\right)H^{*}\right)}^{2}}\right)}^{2}-4\\ \left(rd-2adH^{*}+\frac{2c\left(2aH^{*}-r\right)P^{*}}{b+\left(1-m\right)H^{*}}-\frac{bd\left(1-m\right)P}{{\left(b+\left(1-m\right)H\right)}^{2}}+\frac{c\left(1-m\right)\left(2b+\left(1-m\right)H^{*}\right){\left(P^{*}\right)}^{2}}{{\left(b+\left(1-m\right)H^{*}\right)}^{3}}\right)<0, \end{array}$$

(29) $$\begin{array}{l} r+d-2aH^{*}-\frac{2bcP^{*}+\left(2cH^{*}+b\right)\left(1-m\right)P^{*}}{{\left(b+\left(1-m\right)H^{*}\right)}^{2}}+\tau \\ \left(rd-2adH^{*}+\frac{2c\left(2aH^{*}-r\right)P^{*}}{b+\left(1-m\right)H^{*}}-\frac{bd\left(1-m\right)P}{{\left(b+\left(1-m\right)H\right)}^{2}}+\frac{c\left(1-m\right)\left(2b+\left(1-m\right)H^{*}\right){\left(P^{*}\right)}^{2}}{{\left(b+\left(1-m\right)H^{*}\right)}^{3}}\right)=0. \end{array}$$

Equation (29) is equivalent to

(30) $$B_{1}{\left(m-1\right)}^{3}+B_{2}{\left(m-1\right)}^{2}+B_{3}\left(m-1\right)+B_{4}=0,$$

In which

(31) $$\begin{array}{cc} B_{1}= & \left(2ad\tau +2a\right){\left(H^{*}\right)}^{4}-\left(r+d+\tau dr\right){\left(H^{*}\right)}^{3},\\ B_{2}= & \left(6\tau abd+6ab+4\tau acP^{*}\right){\left(H^{*}\right)}^{3}-\left(3br+3bd-2cP^{*}+3\tau bdr-2\tau crP^{*}\right){\left(H^{*}\right)}^{2}\\ & +\left[\tau bdP^{*}+bP^{*}-\tau c{\left(P^{*}\right)}^{2}\right]H^{*},\\ B_{3}= & \left(6ab^{2}+6\tau ab^{2}d-8\tau abcP^{*}\right){\left(H^{*}\right)}^{2}+\left(4bcP^{*}-3b^{2}r-3b^{2}d-3\tau b^{2}dr+4\tau bcrP^{*}\right)\\ & H^{*}+b^{2}P^{*}+\tau b^{2}dP^{*}-2\tau bc{\left(P^{*}\right)}^{2},\\ B_{4}= & \left(2ab^{3}+2\tau ab^{3}d-4\tau ab^{2}cP^{*}\right)H^{*}-b^{3}r-b^{3}d+2b^{2}cP^{*}-\tau b^{3}dr+2\tau b^{2}crP^{*}. \end{array}$$

According to the Caldano formula, the above one-dimensional cubic Equation (30) can be equated to

(32) $${\left(m-1\right)}^{3}+l_{1}\left(m-1\right)+l_{2}=0,$$

Then, the following can be obtained:

(33) $$m=m_{0}=\sqrt[{3}]{-\frac{l_{2}}{2}+\sqrt{\frac{{l_{1}}^{3}}{27}+\frac{{l_{2}}^{2}}{4}}}+\sqrt[{3}]{-\frac{l_{2}}{2}-\sqrt{\frac{{l_{1}}^{3}}{27}+\frac{{l_{2}}^{2}}{4}}}+1$$

In which

(34) $$\begin{array}{l} l_{1}=\frac{3B_{3}-B_{2}}{3A_{1}},\\ l_{2}=\frac{{B_{2}}^{3}-3B_{2}B_{3}+9{B_{1}}^{2}B_{4}}{9{B_{1}}^{3}}. \end{array}$$

When the parameter conditions satisfy (28) and (33), Map (7) can translate the fixed point $\left(H^{*},\;P^{*}\right)$ to the origin through the transformation $w=H-H^{*}$ and $z=P-P^{*}$. Then, using the Taylor expansion, we can obtain

(35) $$\left(\begin{array}{l} w\\ z \end{array}\right)\to \left(\begin{array}{l} a_{11}w+a_{12}z+\frac{a_{14}}{2}w^{2}+a_{15}wz+\frac{a_{19}}{6}w^{3}+\frac{a_{110}}{2}w^{2}z+O\left({\left(\left|\left.w\right|\right.+\left|\left.z\right|\right.\right)}^{4}\right)\\ a_{21}w+a_{22}z+\frac{a_{24}}{2}w^{2}+a_{25}wz+\frac{a_{28}}{2}z^{2}+\frac{a_{210}}{6}w^{3}+\frac{a_{211}}{2}w^{2}z+\frac{a_{213}}{2}wz^{2}\\ +O\left({\left(\left|\left.w\right|\right.+\left|\left.z\right|\right.\right)}^{4}\right) \end{array}\right)$$

In which the coefficients are all given in Equation (20) with replacements.

The two eigenvalues of Map (35) at the fixed point $\left(0,\;0\right)$ are also conjugate complexes of Mode 1, denoted by

(36) $$\lambda \left(m_{0}\right),\bar{\lambda }\left(m_{0}\right)=-\frac{p\left(m_{0}\right)}{2}\pm i^{\prime }\frac{\sqrt{4q\left(m_{0}\right)-p^{2}\left(m_{0}\right)}}{2}=\alpha \pm i^{\prime }\beta$$

where $i^{’}=\sqrt{-1}$, $p\left(m\right)$ and $q\left(m\right)$ are described in Equations (9) and (10). The value in Equation (33) is obtained as $\left|\lambda \right|=\sqrt{q^{2}\left(m_{0}\right)}=1$ by substituting it into Equation (36). In addition, the condition to generate the Neimark–Sacker bifurcation is also required to be satisfied.

(37) $$\begin{array}{cc} d & =\frac{d\left|\lambda \left(m\right)\right|}{dm}{|}_{m=m_{0}}\\ & =\tau \frac{b^{2}P^{*}-2bcP^{*}H^{*}-\left(2cH^{*}+b\right)\left(1-m\right)P^{*}H^{*}}{{\left[b+\left(1-m\right)H^{*}\right]}^{3}}+\tau^{2}\frac{2c\left(2aH^{*}-r\right)P^{*}H^{*}}{{\left[b+\left(1-m\right)H^{*}\right]}^{2}}\\ & +\tau^{2}\frac{b^{2}dP^{*}-bd\left(1-m\right)P^{*}H^{*}}{{\left[b+\left(1-m\right)H^{*}\right]}^{3}}-\tau^{2}\frac{2b^{2}c{\left(P^{*}\right)}^{2}-2bc\left(1-m\right){\left(P^{*}\right)}^{2}H^{*}-c{\left(1-m\right)}^{2}{\left(P^{*}\right)}^{2}{\left(H^{*}\right)}^{2}}{{\left[b+\left(1-m\right)H^{*}\right]}^{4}}\\ & \neq 0, \end{array}$$

(38) $${\left(\lambda \left(\tau_{0}\right)\right)}^{\theta }\neq 1,\theta =1,2,3,4.$$

From Condition (38), it follows that

(39) $$\tau \left[2aH^{*}-r-d+\frac{2bcP^{*}+\left(2cH^{*}+b\right)\left(1-m_{0}\right)P^{*}}{{\left(b+\left(1-m_{0}\right)H^{*}\right)}^{2}}\right]\neq 2,3.$$

Next, in order to study the canonical type of Map (35), we apply the following reversible transformation to Map (35)

(40) $$\left(\begin{array}{l} w\\ z \end{array}\right)=\left(\begin{array}{cc} a_{12} & 0\\ \alpha -a_{11} & -\beta \end{array}\right)\left(\begin{array}{l} \tilde{w}\\ \tilde{z} \end{array}\right).$$

Then, Map (35) transforms to

(41) $$\left(\begin{array}{l} \tilde{w}\\ \tilde{z} \end{array}\right)\to \left(\begin{array}{cc} \alpha & -\beta \\ \beta & \alpha \end{array}\right)\left(\begin{array}{l} \tilde{w}\\ \tilde{z} \end{array}\right)+\frac{1}{a_{12}\beta }\left(\begin{array}{l} G_{1}\left(\tilde{w},\tilde{z}\right)\\ G_{2}\left(\tilde{w},\tilde{z}\right) \end{array}\right)$$

In which

(42) $$\begin{array}{c} G_{1}\left(\tilde{w},\tilde{z}\right)=a_{12}\beta {\tilde{w}}^{2}\left[\frac{a_{12}a_{14}}{2}+a_{15}\left(\alpha -a_{11}\right)\right]-a_{12}a_{15}\beta^{2}\tilde{w}\tilde{z}-\frac{a_{12}^{2}a_{110}\beta^{2}}{2}{\tilde{w}}^{2}\tilde{z}\\ +a_{12}^{2}\beta {\tilde{w}}^{3}\left[\frac{a_{12}a_{19}}{2}+\frac{a_{110}}{2}\left(\alpha -a_{11}\right)\right]+O\left({\left(\left|\tilde{w}\right|+\left|\tilde{z}\right|\right)}^{4}\right), \end{array}$$

(43) $$\begin{array}{cc} G_{2}\left(\tilde{w},\tilde{z}\right) & =a_{12}{\tilde{w}}^{2}\left[\left(a_{15}-\frac{a_{25}}{2}\right){\left(\alpha -a_{11}\right)}^{2}+a_{12}\left(\frac{a_{14}}{2}-a_{28}\right)\left(\alpha -a_{11}\right)-\frac{a_{12}^{2}a_{24}}{2}\right]\\ & -\frac{a_{12}a_{25}}{2}\beta^{2}{\tilde{z}}^{2}+a_{12}\beta \tilde{w}\tilde{z}\left[\left(a_{25}-a_{15}\right)\left(\alpha -a_{11}\right)+a_{12}a_{28}\right]-\frac{a_{12}^{2}a_{213}\beta^{2}}{2}\tilde{w}{\tilde{z}}^{2}\\ & +a_{12}^{2}{\tilde{w}}^{3}\left[a_{12}\left(\frac{a_{19}}{6}-\frac{a_{221}}{2}\right)\right.\left(\alpha -a_{11}\right)-\frac{a_{12}^{2}a_{210}}{6}+\left(\frac{a_{110}}{2}-\frac{a_{213}}{2}\right)\left.{\left(\alpha -a_{11}\right)}^{2}\right]\\ & +a_{12}^{2}\beta {\tilde{w}}^{2}\tilde{z}\left[\left(a_{213}-\frac{a_{110}}{2}\right)\left(\alpha -a_{11}\right)\right.+\left.\frac{a_{12}a_{211}}{2}\right]+O\left({\left(\left|\tilde{w}\right|+\left|\tilde{z}\right|\right)}^{4}\right). \end{array}$$

Calculate $G_{1}\left(\tilde{w,}\;\tilde{z}\right)$ and $G_{2}\left(\tilde{w,}\;\tilde{z}\right)$ as the second- and third-order derivatives at $\tilde{w}=0$ and $\tilde{z}=0$, i.e.,

(44) $$G_{1\tilde{w}\tilde{w}}=2a_{12}\beta \left[\frac{a_{12}a_{14}}{2}+a_{15}\left(\alpha -a_{11}\right)\right],\;G_{1\tilde{w}\tilde{z}}=-a_{12}a_{15}\beta^{2},\;G_{1\tilde{z}\tilde{z}}=0,\;G_{1\tilde{w}\tilde{w}\tilde{w}}=6a_{12}^{2}\beta \left[\frac{a_{12}a_{19}}{6}+\frac{a_{110}\left(\alpha -a_{11}\right)}{2}\right],\;G_{1\tilde{w}\tilde{w}\tilde{z}}=-a_{12}^{2}a_{110}\beta^{2},\;G_{1\tilde{w}\tilde{z}\tilde{z}}=G_{1\tilde{z}\tilde{z}\tilde{z}}=0,\;G_{2\tilde{w}\tilde{w}}=2a_{12}\left[\left(a_{15}-\frac{a_{25}}{2}\right){\left(\alpha -a_{11}\right)}^{2}+a_{12}\left(\frac{a_{14}}{2}-a_{28}\right)\left(\alpha -a_{11}\right)-\frac{a_{12}^{2}a_{24}}{2}\right],\;G_{2\tilde{w}\tilde{z}}=a_{12}\beta \left[\left(a_{25}-a_{15}\right)\left(\alpha -a_{11}\right)+a_{12}a_{28}\right],\;G_{2\tilde{z}\tilde{z}}=-a_{12}a_{25}\beta^{2},\;G_{2\tilde{w}\tilde{w}\tilde{w}}=6a_{12}^{2}\left[a_{12}\left(\frac{a_{19}}{6}-\frac{a_{211}}{2}\right)\right.\left(\alpha -a_{11}\right)-\frac{a_{12}^{2}a_{210}}{6}+\left(\frac{a_{110}}{2}-\frac{a_{213}}{2}\right)\left.{\left(\alpha -a_{11}\right)}^{2}\right],\;G_{2\tilde{w}\tilde{w}\tilde{z}}=2a_{12}^{2}\beta \left[\left(a_{213}-\frac{a_{110}}{2}\right)\left(\alpha -a_{11}\right)\right.+\left.\frac{a_{12}a_{211}}{2}\right],\;G_{2\tilde{w}\tilde{z}\tilde{z}}=-a_{12}^{2}a_{213}\beta^{2},\;G_{2\tilde{z}\tilde{z}\tilde{z}}=0.$$

In order for Map (41) to undergo a Neimark–Sacker bifurcation near $\left(0,\;0\right)$, the following determinant needs to be satisfied that it is not zero:

(45) a0=−Re1−2λ¯λ¯21−λξ11ξ20−12ξ112−ξ022+Reλ¯ξ21

In which

(46) $$\xi_{11}=\frac{1}{4}\left[\left(G_{1\tilde{w}\tilde{w}}+G_{1\tilde{z}\tilde{z}}\right)+i\left(G_{2\tilde{w}\tilde{w}}+G_{2\tilde{z}\tilde{z}}\right)\right],\;\xi_{20}=\frac{1}{8}\left[\left(G_{1\tilde{w}\tilde{w}}-G_{1\tilde{z}\tilde{z}}+2G_{2\tilde{w}\tilde{z}}\right)+i\left(G_{2\tilde{w}\tilde{w}}-G_{2\tilde{z}\tilde{z}}-2G_{1\tilde{w}\tilde{z}}\right)\right],\;\xi_{02}=\frac{1}{8}\left[\left(G_{1\tilde{w}\tilde{w}}-G_{1\tilde{z}\tilde{z}}-2G_{2\tilde{w}\tilde{z}}\right)+i\left(G_{2\tilde{w}\tilde{w}}-G_{2\tilde{z}\tilde{z}}+2G_{1\tilde{w}\tilde{z}}\right)\right],\;\xi_{21}=\frac{1}{16}\left[\left(G_{1\tilde{w}\tilde{w}\tilde{w}}+G_{1\tilde{w}\tilde{z}\tilde{z}}+G_{2\tilde{w}\tilde{w}\tilde{z}}+G_{2\tilde{z}\tilde{z}\tilde{z}}\right)+i\left(G_{2\tilde{w}\tilde{w}\tilde{w}}+G_{2\tilde{w}\tilde{z}\tilde{z}}-G_{1\tilde{w}\tilde{w}\tilde{z}}-G_{1\tilde{z}\tilde{z}\tilde{z}}\right)\right].$$

Then, bringing Equation (46) into Condition (45), we get

(47) $$\begin{array}{cc} a_{0} & =-\frac{1}{A_{0}}\left\{M_{0}\right.\left[\left(G_{1\tilde{w}\tilde{w}}+G_{1\tilde{z}\tilde{z}}\right)\left(G_{1\tilde{w}\tilde{w}}-G_{1\tilde{z}\tilde{z}}+2G_{2\tilde{w}\tilde{z}}\right)-\left(G_{2\tilde{w}\tilde{w}}+G_{2\tilde{z}\tilde{z}}\right)\left(G_{2\tilde{w}\tilde{w}}-G_{2\tilde{z}\tilde{z}}-2G_{1\tilde{w}\tilde{z}}\right)\right]\\ & +\left.N_{0}\left[\left(G_{2\tilde{w}\tilde{w}}+G_{2\tilde{z}\tilde{z}}\right)\left(G_{1\tilde{w}\tilde{w}}-G_{1\tilde{z}\tilde{z}}+2G_{2\tilde{w}\tilde{z}}\right)-\left(G_{1\tilde{w}\tilde{w}}+G_{1\tilde{z}\tilde{z}}\right)\left(G_{2\tilde{w}\tilde{w}}-G_{2\tilde{z}\tilde{z}}-2G_{1\tilde{w}\tilde{z}}\right)\right]\right\}\\ & -\frac{1}{32a_{12}^{2}\beta^{2}}\left[{\left(G_{1\tilde{w}\tilde{w}}+G_{1\tilde{z}\tilde{z}}\right)}^{2}+{\left(G_{2\tilde{w}\tilde{w}}+G_{2\tilde{z}\tilde{z}}\right)}^{2}\right]-\frac{1}{64a_{12}^{2}\beta^{2}}\left[{\left(G_{1\tilde{w}\tilde{w}}-G_{1\tilde{z}\tilde{z}}-2G_{2\tilde{w}\tilde{z}}\right)}^{2}\right.\\ & \left.+{\left(G_{2\tilde{w}\tilde{w}}-G_{2\tilde{z}\tilde{z}}+2G_{1\tilde{w}\tilde{z}}\right)}^{2}\right]+\frac{1}{16a_{12}\beta }\left[\alpha \left(G_{1\tilde{w}\tilde{w}\tilde{w}}+G_{1\tilde{w}\tilde{z}\tilde{z}}+G_{2\tilde{w}\tilde{w}\tilde{z}}+G_{2\tilde{z}\tilde{z}\tilde{z}}\right)\right.+\\ & \left.\beta \left(G_{2\tilde{w}\tilde{w}\tilde{w}}+G_{2\tilde{w}\tilde{z}\tilde{z}}-G_{1\tilde{w}\tilde{w}\tilde{z}}-G_{1\tilde{z}\tilde{z}\tilde{z}}\right)\right]\\ & \neq 0, \end{array}$$

In which

$$A_{0}=32a_{12}^{2}\beta^{2}\left[{\left(1-\alpha \right)}^{2}+\beta^{2}\right]\;M_{0}=\left(1-3\alpha +2\alpha^{2}-2\beta^{2}\right)\left(\alpha^{2}-\beta^{2}\right)+\left(6\alpha -8\alpha^{2}\right)\beta^{2}\;N_{0}=2\alpha \beta \left[\left(1-\alpha \right)\left(1-2\alpha \right)-2\beta^{2}\right]-\beta \left(3-4\alpha \right)\left(\alpha^{2}-\beta^{2}\right)$$

**Theorem** **3.** *In the discrete predator–prey system, a Neimark–Sacker bifurcation occurs at the fixed point* $\left(H^{*},\;P^{*}\right)$ *if conditions (28), (33), (37), (39), and (47) are satisfied. When* $a_{0}<0$ *and* d<0 *, the invariant ring of attraction bifurcates at* $m<m_{0}$ *; when* $a_{0}>0$ *and* d>0*, the invariant ring of repulsion bifurcates at* $m>m_{0}$*.*

The detailed calculation process of flip bifurcation is shown below. The parameter values are r=1.19,a=0.6,b=0.16,d=0.37,and c=0.42 after calculating the critical value of the occurrence of the Neimark–Sacker bifurcation $m_{0}=0.104355$. At this time, the discriminant values are $d=-0.9219,a_{0}=-0.0498,$ On the basis of Theorem 3, the system undergoes Neimark–Sacker bifurcation from the attractive invariant ring when $m<m_{0}$. Moreover, from Figure 2b, it can be seen that for the above given parameters, the system first experiences Neimark–Sacker bifurcation then enters a steady state at $m_{0}$.

### 2.3. Numerical Simulation Methods

In this study, we perform various numerical simulations (including bifurcation diagrams, maximum Lyapunov exponential diagrams, phase–plane diagrams, spatial amplitude diagrams, and space–time diagrams) to present the results of parsing analysis intuitively and explore the spatiotemporal dynamics of insect predator–prey systems deeply, as the following:

Bifurcation diagrams are graphs that show the various bifurcation behaviors of the dynamical system, usually with one parameter as the horizontal coordinate and one state variable as the vertical coordinate. In this study, we use moth and spider population densities as vertical coordinates and the refuge effect parameter as horizontal coordinates. Then, we can graphically get the bifurcation information of population dynamics in response to refuge effects.

The maximum Lyapunov exponent diagram is an important indicator used to determine whether a system is chaotic or not, which quantitatively portrays the average degree of convergence of the distance between two neighboring trajectories in the phase–plane space. For discrete systems $H_{t+1}=f(H_{t}$), the Lyapunov exponent is satisfied $\left|\delta \left.H_{t}\right|\approx e^{\lambda t}\left|\delta \right.\left.H_{0}\right|\right.$ where ${\delta H}_{0}$ represents distance between two initial states in the phase–plane space, λ represents the Lyapunov exponent, and ${\delta H}_{t}$ represents that the discrete system evolves from two different initial states to two new states at a distance in the phase–plane space after time *t*. The Lyapunov exponent is given by $\lambda (H_{0})=\underset{t\to \infty }{\lim}\frac{1}{t}\sum_{i=0}^{t-1}ln(\frac{f(H_{i})}{dH}$). When the Lyapunov exponent is greater than 0, the system is in a chaotic state at that time. In addition, the higher the Lyapunov exponent, the more obvious the chaotic characteristics and the stronger the degree of chaos.

Furthermore, in order to clearly present the spatial information of the bifurcation map under different degrees of refuge effect, we draw the phase–plane diagram, which reflects the projection of the numerical solution of the dynamical system onto the phase space or its subspace. This allows us to observe the dynamical states of the system, such as fixed points, invariant rings, chaotic attractors, and so on. However, different chaotic phase–plane diagrams are difficult to recognize, so we apply the following methods to differentiate them.

Time series diagrams demonstrate the change of a state variable over time, where the system variable is the average biomass of prey, which shows the stability of the system. Space–amplitude and space–time diagrams are used to characterize the phase–plane diagram and distinguish chaotic phenomena. The space–amplitude diagram is a superposition of the state quantities of each lattice over a period, in which the horizontal coordinate is the spatial location and the vertical coordinate is the population density. The space–time diagram is a plot of the state quantities of each lattice over time, in which the horizontal coordinate is time and its vertical coordinate is spatial location. In this study, the space–amplitude diagram is drawn by superimposing the first 50 spatial lattices after 60,000 iterations and selecting the state quantities from the last 10,000 iterations [44,45]. The space–time diagram is drawn by superimposing the amplitudes of 100 spatial lattices and plotting the state quantities every 20 iterations after 50,000 iterations [44,45]. From these, we can find the spatiotemporal evolution patterns on chaotic routes of discrete insect predator–prey systems.

All simulations are performed in MATLAB R2016a. By numerical simulation, we observe and discuss the population development dynamics of insect communities. This can then be used to improve pest control and better understand the behavior of insect predator–prey systems. The plotting of these diagrams is based on the theoretical calculations in Section 2.2 and parameter selections in Section 2.4.

### 2.4. Parameter Selection

Moths and spiders form a typical insect interaction, occupying an important position. Moths, due to their destructive power and high fecundity, are well known as destructive pests [46]. Spiders, as predators of insects and other invertebrates, are an important component of natural enemies. So, we use the moth–spider parameter to reflect some of the phenomena of insect communities. In this case, the spiders hunt moths by moving around. In addition, in order to more closely simulate the degree of protection for moths in the refuge, we use all space patches as a refuge in one scenario, and randomly select 50% space patches as the refuge in another scenario. The parameter values used in this study were set referring to the prior literature (Table 1).

Subsequently, according to the literature [47,48,49], we selected two sets of parameters for moth populations with relatively higher and lower growth rates, respectively. We then performed bifurcation analyses based on the two sets of parameters, respectively.

**Table 1:** Moth–spider parameter selection.

	Variables	Interpretation	Range from Literatures	Flip Bifurcation	Neimark–Sacker Bifurcation	Reference
Parameters of predation response and competitive intensity of predator to prey	*a*	Intra-specific competition coefficient of moths	0~0.97	0.8	0.6	Huang et al. [25]
	*b*	Measures the extent to which environment provides protection (moths and spiders)	0.1~1.13	0.9	0.16	Huang et al. [25]
	*c*	The maximum valueof the per capita reduction of moths due to spiders	0.01~0.93	0.44	0.42	Senior et al. [24], Huang et al. [25]
Predator–prey parameter	*r*	Growth rate of moths	1~8	3.37	1.19	Han et al. [47], Hariprita et al. [48]
	*d*	Growth rate of spiders	0.01~0.47	0.33	0.37	Mishra et al. [26,50]
	*m*	The proportion of prey protected by refuge	0~1	0~0.45	0~0.15	-

## 3. Results and Discussion

### 3.1. Flip Bifurcations Produced by Moths with Relatively Higher Growth Rates

**Figure 3:**
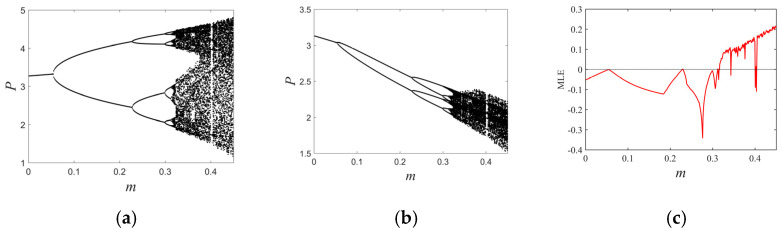
(**a**) Prey flip bifurcation diagram, (**b**) predator flip bifurcation diagram, and (**c**) maximum Lyapunov exponent diagram $\left(a=0.8,\;b=0.9,\;c=0.44,\;r=3.37,\;d=0.33\right)$.

The moth parameter set with higher relative growth rates (see Table 1) produces a flip bifurcation. the flip bifurcation diagrams show that the moth (Figure 3a) and spider (Figure 3b) populations gradually transition from a uniformly fixed state to a chaotic oscillatory state as the parameter m ($m\epsilon \left(0,\left.0.45\right]\right.$) increases. When $m<m^{*}$, the fixed point $\left(H^{*},\;P^{*}\right)$ is stable. However, when $m>m^{*}$, the phenomenon of period-doubling cascades occurs. From the bifurcation diagrams, it can be observed that the increase in the refuge effect led to a greater population density of moths, while the population density of spiders decreased in a short period. The maximum Lyapunov exponential diagram (Figure 3c) shows that as *m* increases beyond the critical value of 0.317, the period-doubling cascades causes the system to exhibit chaotic behavior for the first time. From the maximum Lyapunov exponential diagram, it can be observed that moth and spider population dynamics become chaotic and unpredictable as the refuge effect increases.

**Figure 4:**
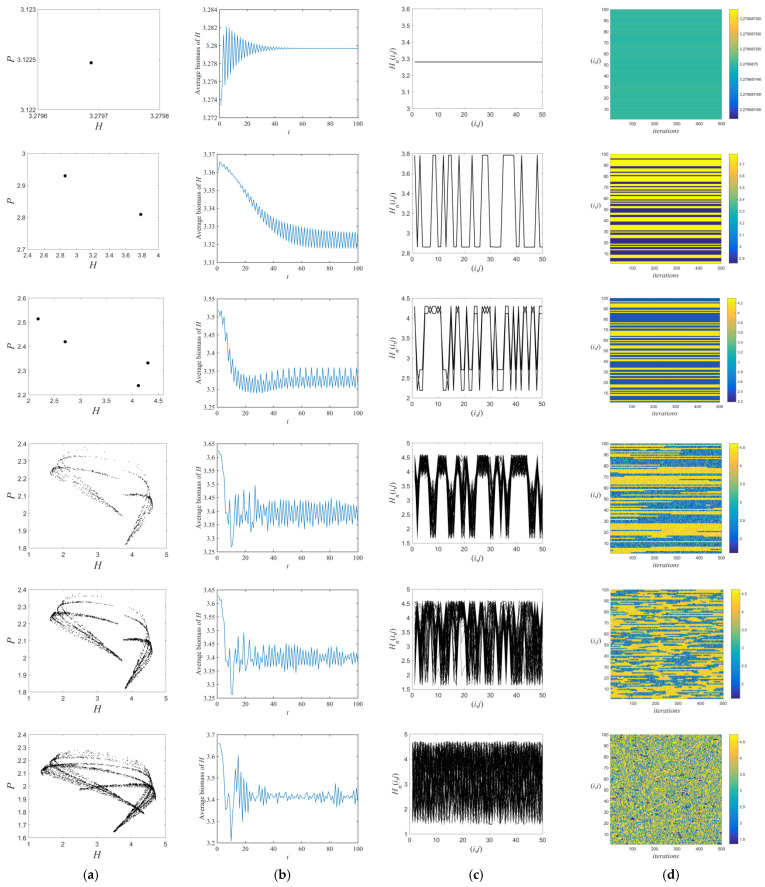
(**a**) Phase–plane diagrams, (**b**) time series diagrams, (**c**) space–amplitude diagrams, and (**d**) space–time diagrams. The parametric conditions are the same as those in Figure 3, except m=0.005,0.1,0.26,0.3755,0.3784,and 0.42, respectively, from the first to the last row. $\left(d_{1}=0.02,\;d_{2}=0.7,\;L=10\;and\;selecting\;of\;100\%\;of\;space\;patches\;as\;the\;refuge\right)$.

**Figure 5:**
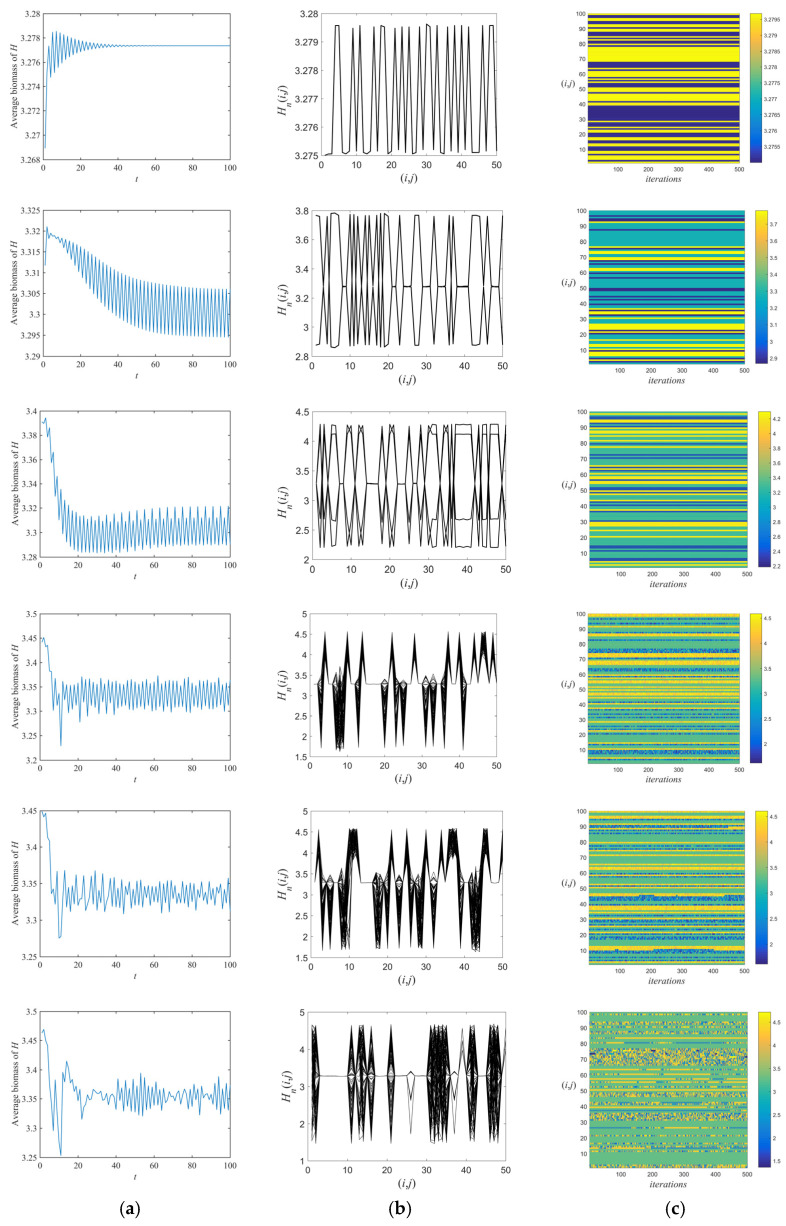
(**a**) Time series diagrams, (**b**) space–amplitude diagrams, and (**c**) space–time diagrams. The parametric conditions are the same as those in Figure 3, except m=0.005,0.1,0.26,0.3755,0.3784,and 0.42, respectively, from the first to the last row. $\left(d_{1}=0.02,\;d_{2}=0.7,\;L=10\;and\;selecting\;of\;50\%\;of\;space\;patches\;as\;the\;refuge\right)$.

The spatiotemporal behavioral development process of the fixed point, the periodic attractor, and the chaotic attractor generated along the chaotic routes under the flip bifurcation is shown in Figure 4 and Figure 5. Ideally, we selected 100% of the space patches as refuge (Figure 4). The time series diagrams (Figure 4b) show that as m increases, the system shifts from a steady state to the oscillatory and unstable state, eventually becoming irregularly chaotic. The spatial amplitude (Figure 4c) shows that the overall moth population in the immobile point state remains constant over time. The system is in a state of cyclic motion at Cycle-2. The orbit of Cycle-4 shows a state of twisting and alternating kinks, in which the positions depend on the initial values. When the system firstly enters the chaotic region ($m=m^{*}$), the spatial amplitude diagram starts to show chaotic and synchronized chaotic regions (m=0.3755), which eventually become difficult to identify.

The space–time diagrams (Figure 4d) in the state of fixed point and periodic attractors show a stable state that is spatially homogeneous, no discontinuities occur, and the pattern remains constant over time. Subsequently, the stable boundary of this system gradually appears fragmentary and defects, and some lattice points even exhibit chaotic behavior. When m increases to 0.3784, the degree of chaos is further strengthened and the spatiotemporal diagrams show multiple stabilized modes transformed by explosive change. It is then difficult to recognize any particular mode in time and space, and the space lattice is almost in a chaotic and turbulent state. The above results indicate that the spatial amplitude and space–time diagrams gradually show chaotic features of disorder and irregularity as the refuge effect increases. In addition, the system gradually develops from a stabilized to fully developed turbulence pattern during this period. It undergoes the frozen random pattern, the defect chaotic diffusion pattern, and the competition intermittency pattern.

More truthfully, we randomly select 50% of the space patches as the refuge (Figure 5). As can be seen in Figure 5a, as m increases, the system gradually becomes unstable and shifts to a chaotic state. Moreover, in comparison to Figure 4c, when the distribution of the refuge is discontinuous, the spatial amplitude changes from a steady state to a state of cyclic motion at the fixed point; the original cyclic motion of the system at Cycle-2 is separated by a number of equilibrium line segments; and the same is true for the Cycle-4 orbital and chaotic regions. The space–time diagrams in the fixed point and periodic attractor states show a wider variety of stable and defect-free frozen bands than in Figure 4d. Under the remaining parameters, it can be seen that new patterns correspond to mixed patterns with frozen bands.

The numerical simulation of flip bifurcation reveals that, as the refuge effect increases to $m^{*}$, the spatiotemporal dynamics of the moths and spiders shift from a steady state to a chaotic state. Starting from m=0.317, the structure of prey populations appears chaotic, which increases the persistence of population survival. These are consistent with previous results “an increase in refuges can increase the density of prey populations, as well as trigger large-scale population explosions [36,51,52]”. In specific conditions, a higher refuge effect contributes to the reproduction of moths. But it may reduce spider population density in a short period, which is consistent with empirical studies [53,54]. Furthermore, through the time series diagrams, the population density of moths gradually tends to stabilize with the development of time when the refuge effect is small. As the refuge effect increases, the population density of moths becomes more irregular and chaotic over time. This suggests that increasing the refuge effect under certain conditions decreases the stability of the system, which is verified by the numerical simulations of the previous theoretical results [55]. Advances on previous studies are made by analyzing the spatial amplitude and space–time diagrams of the system [45,55,56]. We further refine four spatiotemporal development phases (from the frozen random pattern to the defect chaotic diffusion pattern, then the competition intermittency pattern, and finally to the fully developed turbulence pattern) in the chaotic regions and give a means to study spatiotemporal behavior in chaotic population states. Moreover, as the number of refuges decreased, we also found new patterns of the frozen random pattern mixed with the defect chaotic diffusion pattern, competition intermittency pattern, and fully developed turbulence pattern, respectively. Moth populations become stable at regular intervals as the number of refuges decreases under the same refuge effect. These findings offer theoretical guidance (e.g., removing their refuges or setting traps on their escape routes in time) for controlling the reproduction of pests with abundant food sources and high growth rates.

### 3.2. Neimark–Sacker Bifurcation Produced by Moths with Relatively Lower Growth Rates

**Figure 6:**
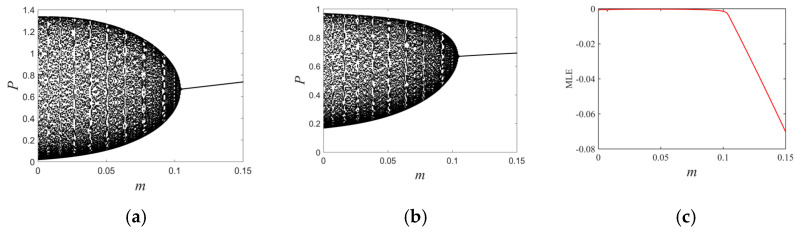
(**a**) Prey flip bifurcation diagram, (**b**) predator flip bifurcation diagram, and (**c**) maximum Lyapunov exponent diagram $\left(a=0.6,\;b=0.16,\;c=0.42,\;r=1.19,\;d=0.37\right)$.

The moth parameter set with relatively lower growth rates (see Table 1) produces a Neimark–Sacker bifurcation showing that the moth (Figure 6a) and spider (Figure 6b) populations change in the interval of $m\epsilon \left(0,\left.0.15\right]\right.$. When $m>m_{0}$, the fixed point $\left(H^{*},\;P^{*}\right)$ is stable. However, the Neimark–Sacker bifurcation occurs when $m<m_{0}$, leads to the destabilization of the moth–spider system. We find that as the refuge effect increases, moths with relatively lower growth rates remain stable at relatively low densities. Moreover, the maximum Lyapunov exponential diagram (Figure 6c) demonstrates that the system stays in an unchaotic state after the Neimark–Sacker destabilization.

**Figure 7:**
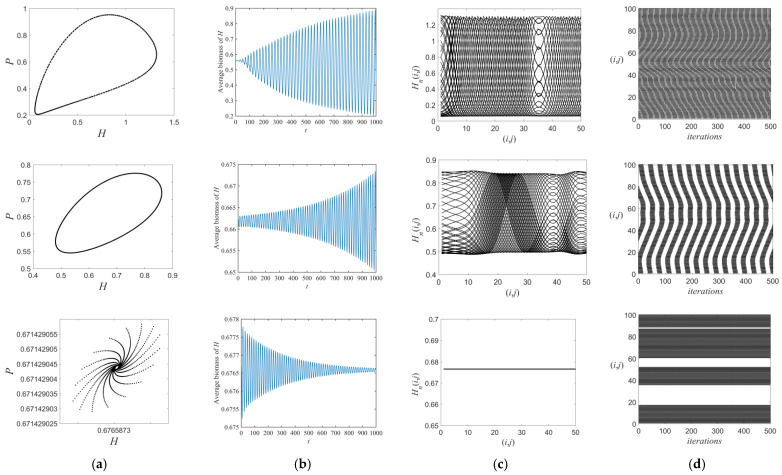
(**a**) Phase–plane diagrams, (**b**) time series diagrams, (**c**) space–amplitude diagrams, and (**d**) space–time diagrams. The parametric conditions are the same as those in Figure 3, except m=0.03,0.1,and 0.11, respectively, from the first to the last row (selecting of 100% of space patches as the refuge).

After the destabilization of the Neimark–Sacker bifurcation, the phase–plane diagrams show that the moth–spider system only exhibits spatiotemporal behavior characterized by the fixed point and the invariant ring (Figure 7a). Ideally, we selected 100% of the space patches as the refuge (Figure 7). As the value of m decreases, the system goes from a stable state to a regularly unstable state (Figure 7b) and undergoes only a frozen random pattern throughout. The corresponding space–amplitude variations display a regular clustered structure. The space–time diagrams show that stable band and bar structures with clear and frozen boundaries do not vary with time. Therefore, the spatiotemporally discrete moth–spider system maintains relative stability both in time and space when the growth rates of moths are relatively lower.

**Figure 8:**
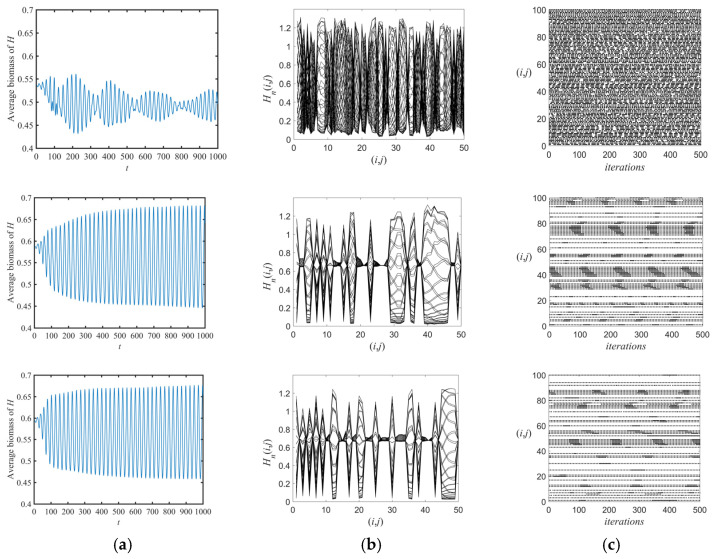
(**a**) Time series diagrams, (**b**) space-amplitude diagrams, and (**c**) space-time diagrams. The parametric conditions are the same with that in Figure 3, except m=0.03,0.1,and 0.11, respectively, from the first to the last row (selecting of 50% of space patches as refuge).

More truthfully, we randomly select 50% of the space patches as the refuge (Figure 8). The time series diagrams in Figure 8a show that as m decreases, the system is regularly but not chaotically unstable. Moreover, compared to Figure 7c, the spatial amplitudes are separated by a number of equilibrium line segments, showing a block of knotty organization. The space–time diagrams show intermittent banding and bar structures with clear and frozen boundaries do not vary with time.

The numerical simulation of Neimark–Sacker bifurcation reveals that the population of moths and spiders progressively transforms from oscillatory behavior to stability with the refuge effect increasing, suggesting that the refuge effect exerts a stabilizing influence on both moth and spider populations, which is in agreement with most previous results [51,55]. The population pattern of the moth system at low growth rates remains in a regularly cycling spatiotemporal state. In addition, as the number of refuge decreases, moth populations become stable at regular intervals under the same refuge effect. Our results complement previous conclusions “increasing refuge effects can trigger population outbreaks” [36,51,52]. These provide theoretical guidance for the management of insect systems. For beneficial insects with a lower growth rate and a preference for specific food sources, appropriately managing the refuge effect is beneficial to maintaining their population stability. However, for pests with low growth rates, we should remove the refuge at times to destabilize their populations.

## 4. Conclusions

Insect predator–prey systems are extremely easy to produce rich dynamic behaviors with, of which the moths–spider system is a typical example. In this study, we clearly unveil the complex spatiotemporal behavior and development law of the insect populations incorporating a refuge effect. Via numerical simulation of two sets of parameters in the moth–spider interaction, we revealed the dynamical behaviors of moths with different growth rates under different levels of the refuge effect and further reflecting the complex mechanisms of insect predator–prey systems, which are shown as follows:

For the moth parameter group with relatively high growth rates, the system produces a flip bifurcation and opens the routes towards chaos. The flip bifurcation appears in the period-2, 4, 8, and 16 bifurcation cascades, a periodic window of period-6 orbits, and chaotic sets. Along these chaotic paths, the system exhibits four distinct spatiotemporal behavior phases: the frozen random pattern, the defective chaotic diffusion pattern, the competition intermittency pattern, and fully developed turbulence pattern. As the refuge effect increases, the initially stable frozen spatiotemporal boundaries break down, then develop into an overall ordered but locally chaotic competitive state, and eventually to chaotic turbulence. And under certain conditions increasing the refuge effect decreases the stability of the system. Moreover, as the number of refuges decreases, we also found new patterns of frozen random pattern mixed with defect chaotic diffusion pattern, competition intermittency pattern, and fully developed turbulence pattern, respectively. This means that the refuge effect can increase the persistence of insect population survival and induce disorganization in spatial and temporal patterns.

For the moth parameter group with relatively low growth rates, the system produces an inverse Neimark–Sacker bifurcation, while it does not produce chaos. This system only appears in the form of fixed points and invariant rings, where the spatiotemporal dynamics always show the frozen random pattern. As the refuge effect increased, moths are able to remain stable at low densities. In addition, as the number of refuge decreases, stabilized space–time structures become more fragmented. This implies that properly reducing the refuge effect can destabilize insect populations, and insect systems under such parameters are always spatially and temporally stable.

In nature, insects often employ various strategies to evade their natural enemy, such as seeking refuges in rocks and bushes, or escaping from predator in time through effective information transmission among insects. In this study, the model explored has practical reference values in insect communities under the refuge effect, and numerical simulations (moths with different growth rates) can yield theoretical support for adjusting refuge effect to destabilize pest populations. Our study suggests that regulating the refuge effect can effectively control pest density, which provides valuable insights for managing invertebrate population dynamics or insect pollination.

## Data Availability

Data is contained within the article.
